# Hypergravity-induced plastic alteration of the vestibulo-sympathetic reflex involves decrease in responsiveness of CAMK2-expressing neurons in the vestibular nuclear complex

**DOI:** 10.1007/s12576-019-00705-5

**Published:** 2019-08-21

**Authors:** Chikara Abe, Yusuke Yamaoka, Yui Maejima, Tomoe Mikami, Hironobu Morita

**Affiliations:** Department of Physiology, Gifu University Graduate School of Medicine, 1-1 Yanagido, Gifu, 501-1194 Japan

**Keywords:** Gravity, Vestibule, Plasticity, Arterial pressure, Optogenetics, Viral vector

## Abstract

The vestibular system contributes to not only eye movement and posture but also the sympathetic response. Plastic alteration of the vestibulo-sympathetic reflex is induced by hypergravity load; however, the mechanism remains unknown. Here, we examined 2 g-induced changing in responsiveness of CAMK2-expressing neurons in the vestibular nucleus complex using optogenetic tools. The excitatory photostimulation of the CAMK2-expressing neurons in the unilateral vestibular nuclear complex induced body tilt to the contralateral side, while inhibitory photostimulation showed the opposite response. Photoactivation of either cell body or the axonal terminal in the rostral ventrolateral medulla showed sympathoexcitation followed by the pressor response. Furthermore, this response was significantly attenuated (49.8 ± 4%) after the 1st day of 2 g loading, and this value was further reduced by the 5th day (22.4 ± 3%), suggesting that 2 g-induced attenuation of the vestibulo-sympathetic reflex involves at least decrease in responsiveness of CAMK2-expressing neurons in the vestibular nuclear complex.

**Electronic supplementary material:**

The online version of this article (10.1007/s12576-019-00705-5) contains supplementary material, which is available to authorized users.

## Introduction

The peripheral gravity sensor, located in the inner ear, consists of two components, namely, the semicircular canals and otolith organs, which detect angular and linear accelerations, respectively. The signals from each sensor are transmitted to the brain, specifically to the vestibular nuclear complex (VNC), through the vestibular nerve [1]. The vestibular system contributes to both eye movement (i.e., the vestibulo-ocular reflex) and posture (i.e., the vestibulo-spinal reflex), and is important for the understanding of the body’s dynamics and kinematics [2]. The vestibular system also participates in the sympathetic nervous response (vestibulo-sympathetic reflex) [3, 4]. Acute gravitational change or galvanic vestibular stimulation (GVS) increases the sympathetic nerve activity in rodents and human subjects [5–7]. The vestibulo-sympathetic reflex may contribute to orthostatic tolerance, which is typically associated with the baroreflex [8, 9]. Furthermore, recent studies have shown that the vestibular system is involved in other physiological functions; muscles and bone [10], food intake [11], circadian rhythm [12], and body temperature regulation [13].

The vestibular system is one of the plastic organs which is easily affected by chronic gravitational changes [14]. The studies using astronauts have reported the decrease in sensitivity of vestibulo-ocular [15], vestibulo-spinal [16], and vestibulo-sympathetic (cardiovascular) reflexes [9, 17], which increase the risk of falls and bone fractures. In addition to microgravity, we demonstrated that these plastic alterations in the vestibulo-sympathetic reflex also occur in rats exposed to hypergravity [5, 18, 19]. When rats were maintained in a hypergravity environment, the sympathetic and cardiovascular responses induced by vestibular stimulation, including free drop and linear acceleration, were attenuated. Although we clarified that attenuation of the vestibulo-sympathetic reflex is due to a decrease in head movement-related phasic inputs to the peripheral vestibular organs [20, 21], the mechanism of the plastic alteration is still unclear.

The morphological and genetic changes in the peripheral vestibular organ are induced by the gravitational change. Exposure to microgravity for 9 days significantly increased total ribbon synapses and sphere-like ribbons in type I and II hair cells in rats [22]. On the other hand, hypergravity load for 1 week showed a decrease in GluR2 mRNA expression in the vestibular ganglion [23]. These findings indicate that gravitational change might influence the responsiveness of neurons in the VNC because these neurons are downstream of the vestibular pathway. Indeed, NR1 mRNA expression in the VNC was decreased by hypergravity load [23]. If this is true, activation of neurons in the VNC-induced sympathetic response might be attenuated in rats which are maintained in the hypergravity environment. To test this hypothesis, we focused on the functional change of the putative excitatory neuron, calcium/calmodulin-dependent protein kinase II (CAMK2)-expressing neurons, in the VNC induced by chronic 2 g loading, and then evaluated whether the sympathoexcitation induced by optogenetic stimulation of these neurons was attenuated.

## Materials and methods

### Animals

The animals used in the present study were maintained in accordance with the “Guiding Principles for Care and Use of Animals in the Field of Physiological Science”, set by the Physiological Society of Japan. The experiments were approved by the Animal Research Committees of Gifu University. Male (*n* = 38) and female (*n* = 32) rats, weighing 220–280 g, were purchased from Chubu Kagaku Shizai.

### Anesthesia and postoperative management

All surgeries were conducted under aseptic conditions, and rats were anesthetized with a mixture of medetomidine hydrochloride (0.3 mg/kg), midazolam (4 mg/kg), and butorphanol tartrate via the intraperitoneal administration (i.p.) route (5 mg/kg). The depth of anesthesia was deemed sufficient when the corneal and hind-paw withdrawal reflexes were absent. Additional anesthetic was administered as necessary (10% of the original dose, i.p.). Body temperature was maintained at 37.0 ± 0.5 °C with a servo-controlled temperature pad. After surgery, rats received postoperative boluses of atipamezole (an α2-adrenergic antagonist, 2 mg/kg), penicillin G potassium (3000 U/kg), and ketoprofen (4 mg/kg) via the subcutaneous administration (s.c.) route. Rats were solely housed to maintain the ferrule on the head. Rats were under a 12:12-h light–dark cycle. The room temperature was maintained at 24 ± 1 °C.

### Injection of the viral vectors into the VNC and optical fiber placement

**Fig. 1:**
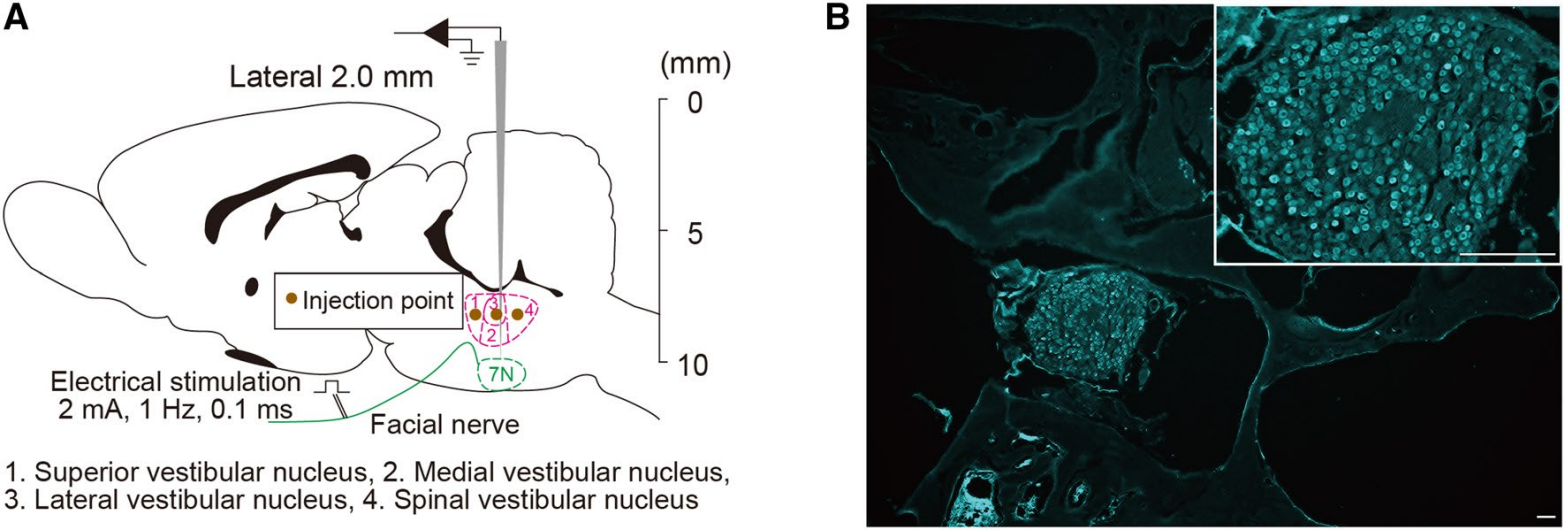
**a** Injection of viral vector to express either ChR2 or eArchT in the CAMK2-expressing neurons in the vestibular nucleus complex (VNC). The facial nucleus, which is found through the evoked potential induced by electrical stimulation of the facial nerve, is regarded as a landmark to find the VNC. **b** Neuronal retrograde tracer (Fluoro-Gold) was injected at the left VNC. Fluoro-gold expression was observed in the vestibular ganglion. Scale bar: 100 μm for main panels and 200 μm for insets

AAV2-CaMK2a-hChR2(H134R)-mCherry, AAV2-CaMK2a-eArchT3.0-EYFP, and AAV2-CaMK2a-mCherry were purchased from the University of North Carolina vector core [obtained courtesy of K. Deisseroth (Stanford University)]. These viral vectors were injected unilaterally into the left VNC, followed by the placement of an optical fiber (Fig. 1a). Using a rat brain atlas, the VNC was located at the dorsal side from the facial motor nucleus. The left mandibular branch of the facial nerve was revealed through a small skin incision for successive electrical stimulation. Thereafter, the rat was placed prone on a stereotaxic apparatus (SR-6M-HT, Narishige). The viral vector was loaded into a 1.2 mm internal diameter glass pipette broken to a 25 μm tip (external diameter) and introduced into the brain through a 1.5 mm diameter hole drilled into the occipital plate caudal to the parieto-occipital suture on the left side. The facial nerve was then stimulated (0.1 ms, 1–300 μA, 1 Hz) to evoke antidromic field potentials within the facial motor nucleus. These field potentials, recorded via the vector-filled pipette, were used to map the facial motor nucleus (FN) and identify the location of the VNC, which resides dorsal to the facial motor nucleus. Three 140 nL injections were performed 3000 μm above the base of the medulla oblongata (determined as the lower limit of the facial field potential). The three injections were separated by 200 μm and were aligned rostrocaudally. Successively, an optical fiber (250 μm core after desheathing; Thorlabs) was inserted 300 μm above the injection site and secured to the skull through a cyanoacrylate adhesive. Prior to the implantation, the optical fibers were glued to a zirconia ferrule (outside diameter 1.25 mm; bore 230 μm; Thorlabs).

### Injection of the fluoro-gold into the VNC or rostral ventrolateral medulla (RVLM)

To examine the projection from the vestibular ganglion to the VNC, neuronal retrograde tracer (1% Fluoro-Gold, Fluorochrome, LLC, CO) was injected into the left VNC using the above mentioned method. After the experiment, decalcifying liquid (Kalkitox, Wako, Tokyo, Japan) was applied to the left labyrinths. The same tracer was injected into the RVLM to examine the projection from the VNC to RVLM. For unilateral administration of vector in the RVLM, three 14 nL injections were made 300 μm above the base of the medulla oblongata (determined as the lower limit of the facial field potential) and at the medial edge of the respiratory column (identified by respiratory synchronous multiunit activity). The three injections were separated by 200 μm and were aligned rostrocaudally.

### Balance test using photostimulation

A blue laser (473 nm; MBL-III-473, Changchun New Industries Optoelectronics Technology Co. Ltd.) and a green laser (532 nm; MBL-III-532, Changchun New Industries Optoelectronics Technology Co. Ltd.) were used to stimulate the ChR2 and eArchT of the neurons located in the VNC, respectively. The light output of each optical fiber was measured with a light meter (PM20A, Thorlabs), and the laser setting required to deliver 10 mW was recorded. Rats were briefly anesthetized with isoflurane while the connection between the implanted fiber optic and the laser delivery system was established. After recovery from the isoflurane inhalation, a rat was placed on a rod located 50 cm above the ground. The parameters related to photostimulation are as follows: duration, 10 ms; frequency, 10, 20, 30, or 40 Hz. Photostimulation was also applied for a duration of 1 s (hold). Body tilt was recorded during photostimulation using a video camera (EX-100F, CASIO), and its degree was calculated using software (https://www.kinovea.org/).

### Measurement of arterial pressure (AP) and renal sympathetic nerve activity (RSNA)

A polyethylene catheter (PE-50; Becton–Dickinson, Sparks, MD) was inserted into the abdominal aorta via the left femoral artery to measure AP. The catheter was exteriorized from the back of the neck. For recording the RSNA, the postganglionic renal sympathetic nerve was isolated through a right or left flank incision, and two stainless-steel electrodes (AS633; Cooner Wire, Chatworth, CA) were placed around it. The nerve and electrodes were covered and fixed with Low Toxicity Silicone Adhesive (Kwik-Sil, World Precision Instruments), and the electrodes were exteriorized at the back of the neck. The catheter was connected to a pressure transducer (MP5200; Baxter, Deerfield, IL) placed at the cardiac level. The signal from the transducer was transmitted to an amplifier (MEG-6108; Nihon Kohden, Tokyo, Japan). The electrodes for recording RSNA were connected to an amplifier (MEG-1200; Nihon Kohden, Tokyo, Japan) equipped with a 50- to 1000-Hz band-pass filter. The output from the amplifier was passed through a gate circuit to subtract baseline noise, and it was rectified by an absolute value circuit. All signals were recorded using an analog-to-digital converter (PowerLab; AD Instruments, New South Wales, Australia) at a rate of 1000 Hz.

### Immunohistochemistry

Rats were euthanized with an overdose of pentobarbital sodium and perfused transcardially with 100 mL of heparinized saline, followed by 150 mL of freshly prepared 4% paraformaldehyde in sodium phosphate buffer (pH 7.4). Subsequently, their brains were extracted and post-fixed at 4 °C for 24–48 h in the same fixative. Transverse sections (40 μm thick) were then cut using a cryotome and stored in a cryoprotectant solution (20% glycerol plus 30% ethylene glycol in 50 mM phosphate buffer, pH 7.4) at − 20 °C. To confirm the expression of either the mCherry or eYFP in the VNC, the following antibodies were used: the mCherry protein was detected with the anti-DsRed (mouse monoclonal, 1:500; sc-390909 AF594; Santa Cruz Biotechnology); the eYFP protein was detected with the anti-GFP (chicken polyclonal, 1:500; GFP-1010; Aves Labs) followed by the Alexa Fluor-488-tagged rabbit anti-chicken antibody (1:200; Jackson ImmunoResearch Laboratories). To examine the c-fos expression, the anti-c-fos antibody (1:1000; Millipore #ABE457; EMD Millipore) was used, followed by the Alexa Fluor-594-tagged donkey anti-rabbit antibody (1:200; Jackson ImmunoResearch Laboratories). For the detection of Tyrosine Hydroxylase (TH)-expressing neurons, the anti-TH antibody (1:1000; Millipore #AB1542; EMD Millipore) was used, followed by the Alexa Fluor-488-tagged donkey anti-sheep antibody (1:200; Jackson ImmunoResearch Laboratories). Thereafter, the brain sections were analyzed using fluorescence microscopy (BZ-X800, KEYENCE). The output levels were adjusted to include all the information-containing pixels, while the balance and contrast were adjusted to reflect the true rendering as much as possible. No other image retouching was performed.

### Vestibular lesion

Sodium arsanilate solution (100 mg/mL) was injected into the bilateral middle-ear cavity (50 μL/ear) for vestibular lesion (VL) under light sedation with isoflurane (Escain, Pfizer). Same volume of the saline was used for the sham operation (Sham). The success of VL was confirmed by observing the swimming behavior of the rats after they were gently placed in a small tub of water. Rats with complete VL were unable to determine the direction in which they had to swim to reach the surface and continued to turn around under water.

### Exposure to the hypergravity environment

The hypergravity environment was induced by centrifugation using a custom-made gondola-type rotating box (Shimadzu). Rats were fed ad libitum, the cages were maintained on a 12:12-h light–dark cycle, and the room temperature was maintained at 24 ± 1 °C. Rats were maintained in groups for the detection of c-fos in C1 neurons; however, rats were individually maintained in the case of the AP and RSNA measurements to avoid damage to the catheter and electrodes.

### Statistical analysis

All the data sets were tested for normality using either the D’Agostino–Pearson omnibus normality or Kolmogorov–Smirnov tests. Equal variances were examined successively through the Brown–Forsythe test. If the criteria of normality and equal variance were satisfied, the statistical significance was evaluated using either one- or two-way ANOVA, followed by the Tukey–Kramer, Dunnett or Bonferroni tests. All values were expressed as mean ± SEM, while the statistical significance was set at *P* < 0.05.

## Results

### CAMK2-expressing neurons in the VNC are involved in balance function

**Fig. 2:**
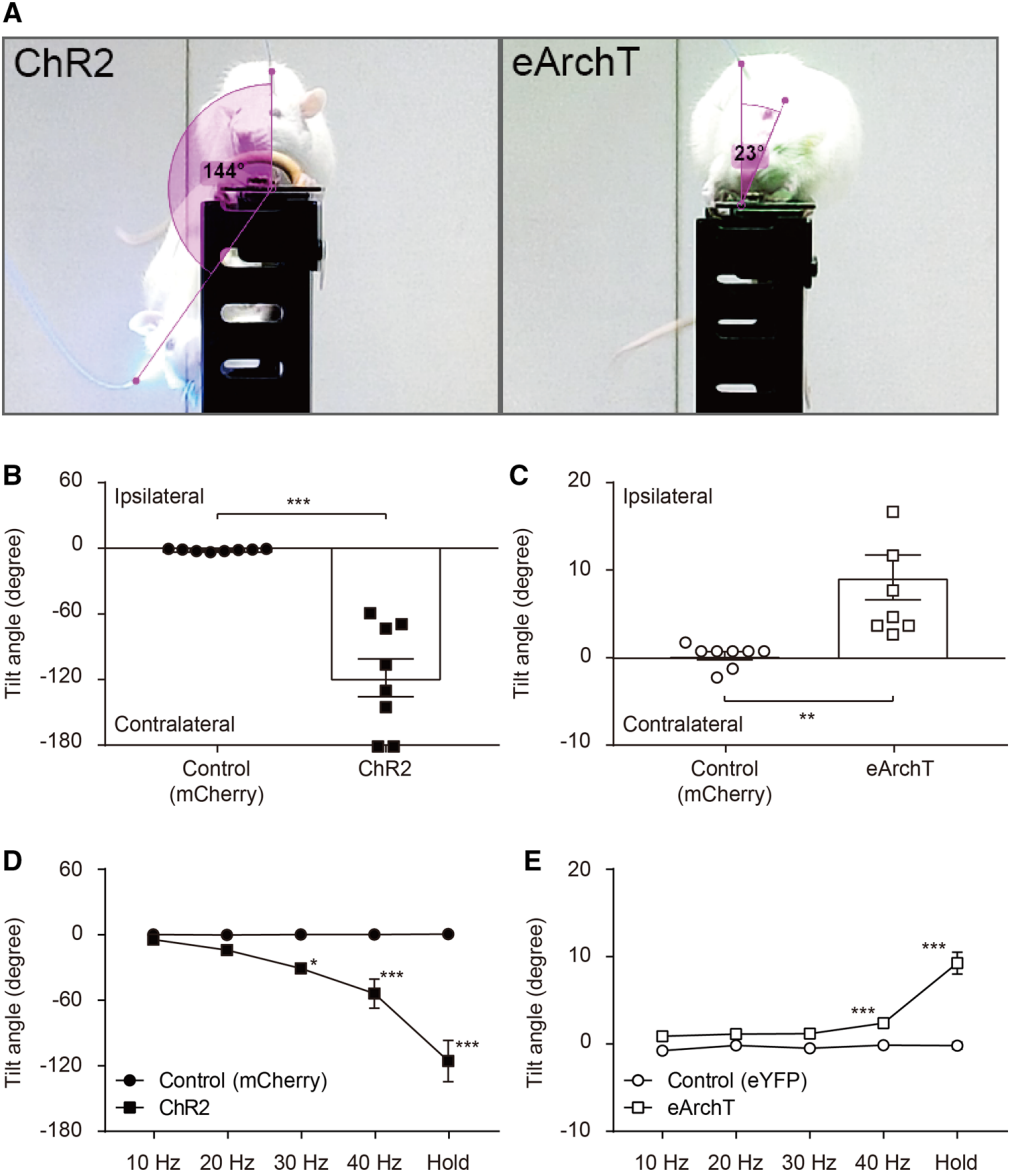
**a** Photostimulation of either the ChR2 (blue) or the eArchT (green) in the CAMK2-expressing neurons located in the vestibular nucleus complex (VNC) induced body tilt. Excitatory photostimulation induced body tilt to the contralateral side while inhibitory photostimulation showed the opposite response. Please see the Video S1. **b**, **c** Summarized data of the changes in body tilt angle induced by excitatory (**b**) or inhibitory (**c**) photostimulation. Statistics: unpaired *t* test; *t*(14) = 6.726, *P* < 0.0001 (**b**) and *t*(14) = 3.446, *P* = 0.0039 (**c**). **d**, **e** Summarized data of the body tilt angle induced by changes in the frequency of excitatory (**d**) or inhibitory (**e**) photostimulation. A two-way ANOVA with Tukey’s multiple comparisons test was applied; *F*(4, 56) = 27.11, *P* < 0.0001 (**d**) and *F*(4, 56) = 29.96, *P* < 0.0001 (**e**). For all statistical analyses, single, double, or triple significance symbols indicate *P* < 0.05, *P* < 0.01, or *P* < 0.001, respectively (color figure online)

**Fig. 3:**
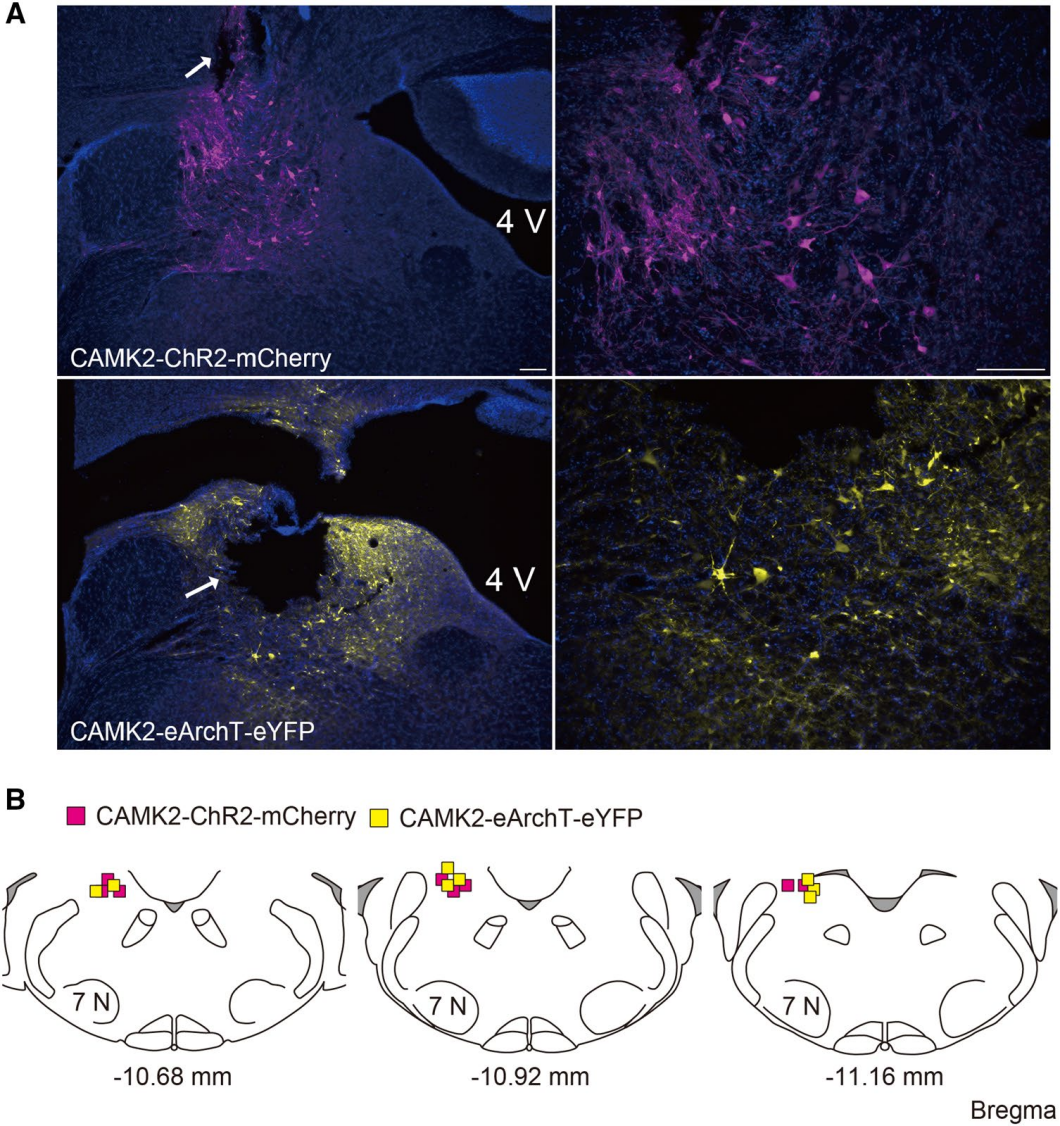
**a** Representative image of either ChR2 or eArchT expression in the CAMK2-expressing neurons in the vestibular nucleus complex (VNC). The arrow indicates the trace of the fiber optic. The scale bar is 100 μm. **b** Location of the fiber optic tips for the excitatory (ChR2) or inhibitory (eArchT) photostimulation in the CAMK2-expressing neurons in the VNC. Distance from caudal to bregma (mm) is shown at lower of each section

To measure the VNC receiving the gravitational inputs through the vestibular nerve, we injected the neuronal retrograde tracer (1% fluoro-gold) into the VNC. Expression of fluoro-gold was observed in the ipsilateral vestibular ganglion 2 days after injection (Fig. 1b). Next, we examined the role of CAMK2-expressing neurons on the balance function. Opsin, ChR, or eArchT was expressed in CAMK2-expressing neurons in the unilateral VNC. We applied photostimulation to these neurons using a blue (473 nm) or green laser (532 nm) in the rat which was placed on the rod. Excitation (blue laser) of the CAMK2-expressing neurons in the VNC induced body tilt to the contralateral side, while the opposite response was observed by neural inhibition (green laser) (Fig. 2a and Video S1). In both cases, a significant increase in tilt angle was observed although the direction of the body tilt was different (Fig. 2b, c). This tilt angle was dependent on the intensity (stimulus frequency) of the photostimulation (Fig. 2d, e). Since we confirmed both the appropriate expression of the opsins, including both ChR2 and eArchT, and the placement of the fiber optics (Fig. 3a, b), it can be concluded that CAMK2-expressing neurons in the VNC participate in the balance function.

### CAMK2-expressing neurons in the VNC are involved in the autonomic response

**Fig. 4:**
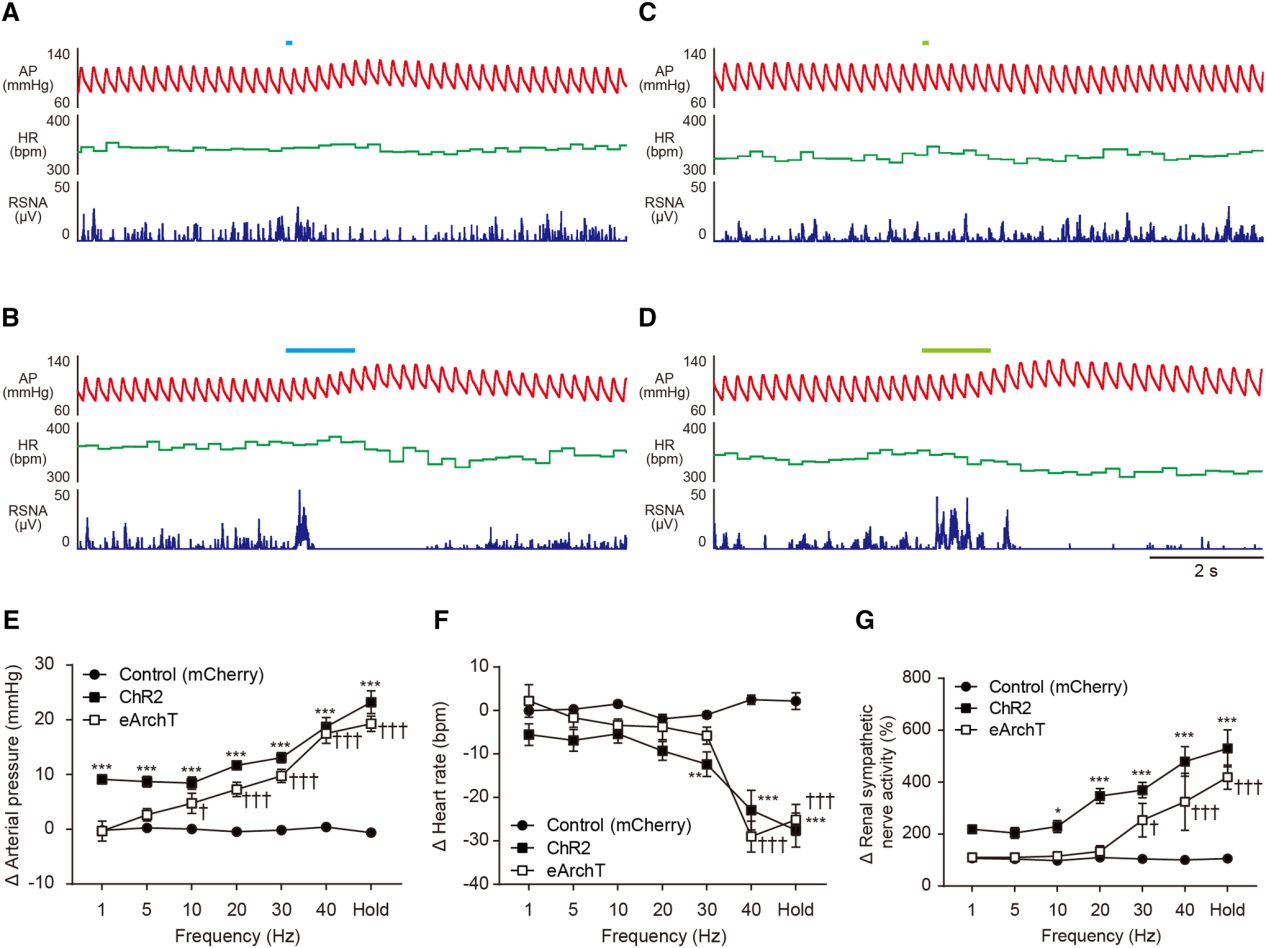
**a**, **b** Effect of excitatory (**a**, **b**) or inhibitory (**c**, **d**) photostimulation of the CAMK2-expressing neurons in the vestibular nucleus complex (VNC) on the arterial pressure (AP), heart rate (HR), and renal sympathetic nerve activity (RSNA) in conscious rats. The duration was 10 ms (**a**, **c**) or 1 s (**b**, **d**). **e**–**g** Summarized data of change in AP (**e**), HR (**f**), and RSNA (**g**) induced by excitatory (ChR2) or inhibitory (eArchT) photostimulation of the CAMK2-expressing neurons in the VNC in conscious rats. For their control, the blue laser (473 nm) was applied to the rats into which the control vector (AAV-CAMK2-mCherry) was injected. The frequency of the photostimulation was varied from 1 to 40 Hz. In this case, the duration of each pulse was 10 ms. The frequency “Hold” means the photostimulation with the duration of 1 s. A two-way ANOVA with Tukey’s multiple comparisons test was applied; *F*(12, 126) = 15.71, *P* < 0.0001 (**e**), *F*(12, 126) = 10.66, *P* < 0.0001 (**f**) and *F*(12, 126) = 4.174, *P* < 0.0001 (**g**). * or ^†^ vs. Control (mCherry). For all statistical analyses, single or triple significance symbols indicate *P* < 0.05 or *P* < 0.001, respectively

**Fig. 5:**
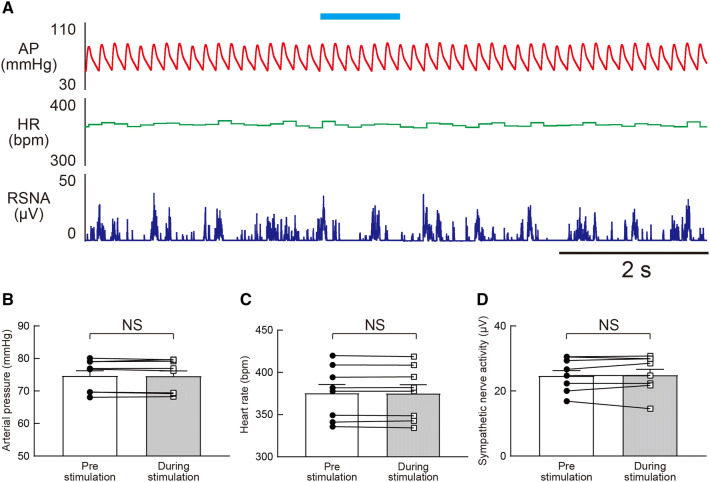
**a** Effect of excitatory photostimulation of CAMK2-expressing neurons in the vestibular nucleus complex (VNC) on the arterial pressure (AP), heart rate (HR), and renal sympathetic nerve activity (RSNA) in an anesthetized rat. **b**–**d** Summarized data of the change in AP (**b**), HR (**c**), and RSNA (**d**) induced by excitatory (ChR2) photostimulation of CAMK2-expressing neurons in the VNC in anesthetized rats. The duration of the photostimulation was 1 s

We investigated whether the CAMK2-expressing neurons in the VNC can induce a sympathetic and a cardiovascular response in rats. In the excitation of CAMK2-expressing neurons in the VNC, photostimulation for 10 ms showed an increase in the AP (Fig. 4a). This response was greater upon photostimulation for 1 s, and the RSNA became prominent (Fig. 4b). On the other hand, inhibition for 10 ms of the CAMK2-expressing neurons in the VNC did not show any response; however, stronger photostimulation (1 s) showed an increase in both AP and RSNA (Fig. 4c, d). In these responses, both excitation and inhibition of CAMK2-expressing neurons in the VNC showed a photostimulation intensity-dependent (stimulus frequency-dependent) increase in responses (Fig. 4e, g). With regard to the heart rate (HR) response, stronger photostimulation induced bradycardia (Fig. 4f). Interestingly, the photostimulation-induced (1 s) responses including AP, HR, and RSNA were not observed in the anesthetized rats (Fig. 5a–d), suggesting that autonomic responses induced by vestibular stimulation require a conscious condition.

**Fig. 6:**
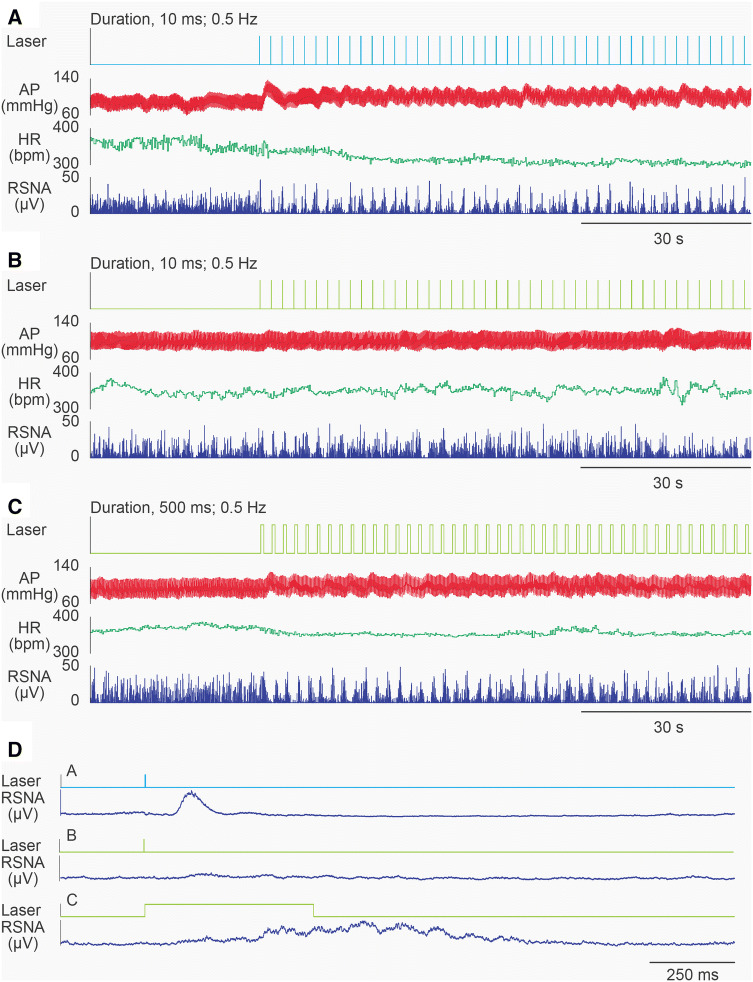
Representative data of excitatory (ChR2, **a**) or inhibitory (eArchT, **b**, **c**) photostimulation of the CAMK2-expressing neurons in the vestibular nucleus complex (VNC). Photostimulation produced a robust activation of renal sympathetic nerve activity (RSNA) in conscious rats. The frequency of the photostimulation is 0.5 Hz. The duration of the photostimulation is 10 ms (**a**, **b**) or 500 ms (**c**). **d** Averaged data of the evoked potential in RSNA induced by excitatory or inhibitory photostimulation of the CAMK2-expressing neurons in the VNC. The data were superimposed 100 times

The present study demonstrated that not only the excitation but also the inhibition of the CAMK2-expressing neurons in the VNC resulted in an increase in RSNA. To investigate further, we measured the evoked potential of the renal sympathetic nerve induced by photostimulation of CAMK2-expressing neurons in the VNC in conscious rats. The nerve activity evoked by low-frequency photostimulation (0.5 Hz) was determined by constructing peristimulus waveform averages of the half-wave rectified signal (100 sweeps) (Fig. 6a–c). Excitatory photostimulation for a duration of 10 ms resulted in an evoked potential with a sharp peak (Fig. 6d); the onset latency was 75.1 ± 1.7 ms (*n* = 8). However, there was no evoked potential of the renal sympathetic nerve upon inhibitory photostimulation (Fig. 6d). Even upon inhibitory photostimulation, the evoked potential, which had a wide skirt form, was observed when the duration of the stimulation was set to 500 ms (Fig. 6d).

### Neural projection from the VNC to the RVLM

**Fig. 7:**
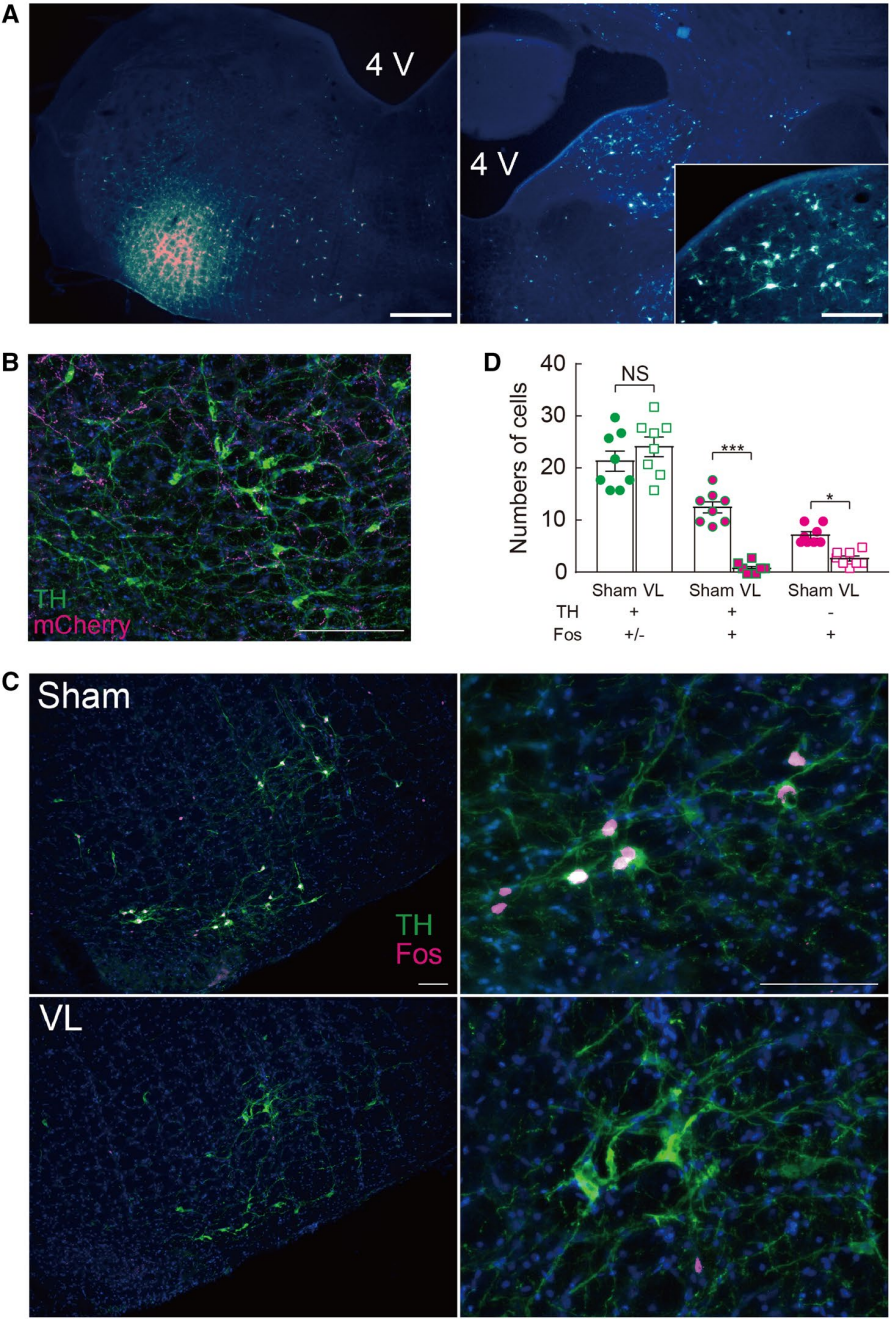
**a** Neuronal retrograde tracer (Fluoro-Gold) was injected at the left rostral ventrolateral medulla (RVLM). Fluoro-gold expression was observed in the vestibular nucleus complex (VNC). Scale bar: 200 μm for main panels and 100 μm for insets. **b** Axonal terminal of the neurons in the VNC at the RVLM. Viral vector, AAV-CAMK2-mCherry, was injected at the VNC. The scale bar is 100 μm. **c** c-fos expression in C1 neurons induced by exposure to hypergravity environment (2 g) for 90 min in rats with vestibular lesion (VL) and their sham operation (Sham). The scale bar is 100 μm. **d** Quantitative analysis of c-fos expression induced by 2 g loading in Sham and VL rats. Statistics: unpaired *t* test; *t*(14) = 1.017, *P* = 0.3266 [TH(+) Fos(±)], *t*(14) = 10.26, *P* < 0.0001 [TH(+) Fos(+)], and *t*(14) = 5.713, *P* < 0.0001 [TH(−) Fos(+)]

To examine the neural circuit of the vestibulo-sympathetic reflex, we injected neuronal retrograde tracer (1% fluoro-gold) into the RVLM (Fig. 7a). Bilateral neurons in the VNC were labeled, suggesting that the neurons in the VNC project into the RVLM bilaterally. Furthermore, the terminal end of the neurons in the VNC was observed in the RVLM; some terminals were observed alongside the C1 neurons (Fig. 7b). Next, we examined whether the vestibular stimulation activates C1 neurons in the RVLM. Exposure to a hypergravity environment for 90 min showed expression of c-fos in C1 neurons in the RVLM; this was significantly suppressed by VL (Fig. 7c, d). In non-C1 neurons in the RVLM, c-fos expression was also significantly suppressed by VL (Fig. 7d).

**Fig. 8:**
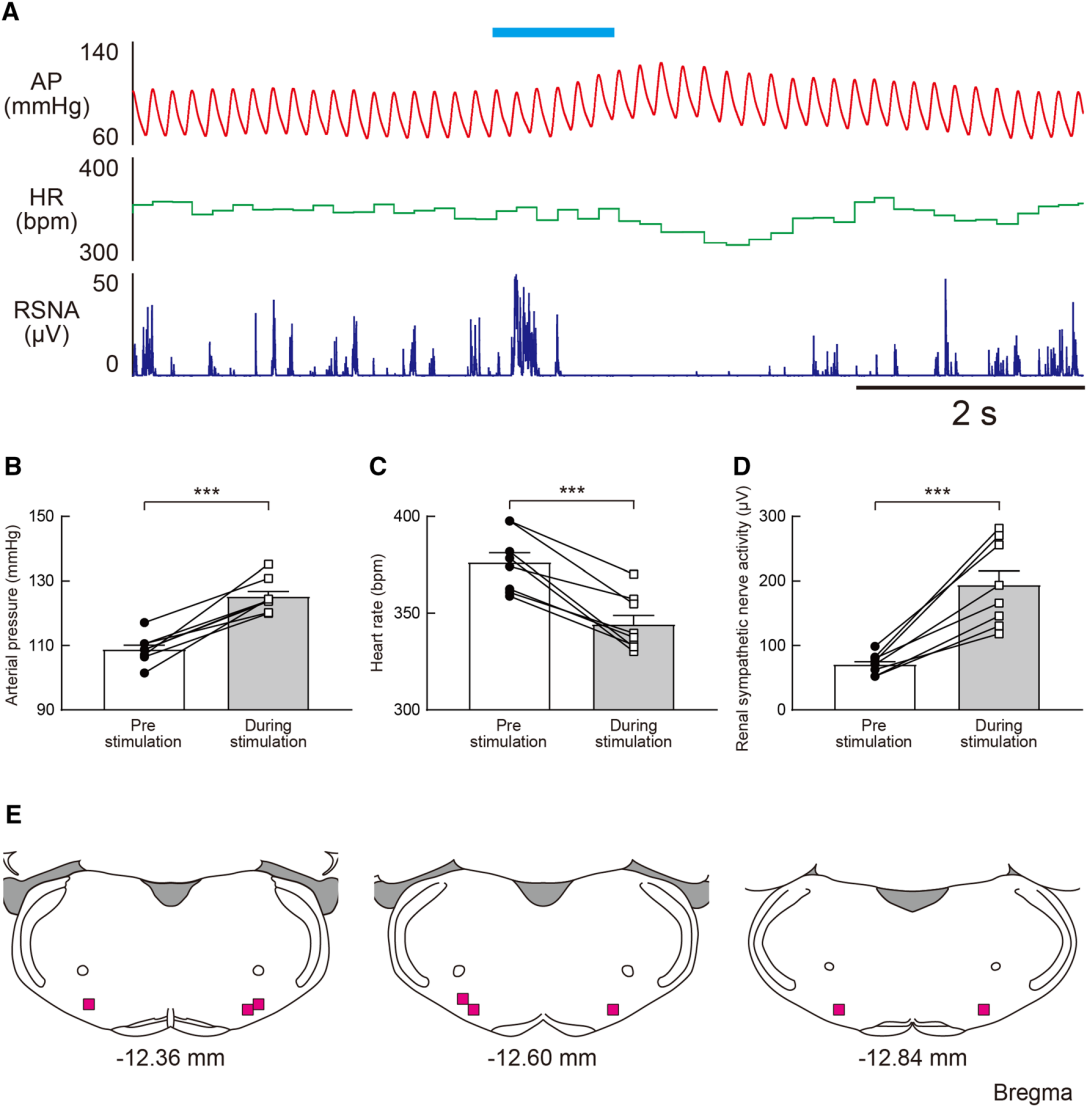
**a** Effect of excitatory photostimulation at the axonal terminal in the rostral ventrolateral medulla (RVLM) from the CAMK2-expressing neurons in the vestibular nucleus complex (VNC) on the arterial pressure (AP), heart rate (HR), and renal sympathetic nerve activity (RSNA) in conscious rats. The duration of the photostimulation is 1 s. **b**–**d** Summarized data of the change in AP (**b**), HR (**c**), and RSNA (**d**) induced by excitatory (ChR2) photostimulation at the axonal terminal in the RVLM from CAMK2-expressing neurons in the VNC in conscious rats. The duration of the photostimulation is 1 s. Statistics: paired *t* test; *t*(7) = 7.737 *P* = 0.0001 (**b**), *t*(7) = 7.055 *P* = 0.0002 (**c**), and *t*(7) = 6.375, *P* = 0.0004 (**d**). Triple significance symbol indicates *P* < 0.001. **e** Location of the fiber optic tips for the excitatory (ChR2) photostimulation at the axonal terminal in the RVLM from CAMK2-expressing neurons in the VNC

To examine whether the vestibulo-sympathetic response occurs due to the monosynaptic neural projection from the VNC to the RVLM, we measured sympathetic and cardiovascular responses during excitatory photostimulation of the terminal end of CAMK2-expressing neurons in the VNC which project into the RVLM. An increase in RSNA followed by the pressor response was observed upon photostimulation (1 s) (Fig. 8a). The photostimulation of either the right or left RVLM significantly increased RSNA and AP while also decreasing the HR (Fig. 8b–d). The onset latency of the evoked potential, measured using the same methods as those used to capture the data of Fig. 6, was 53 ± 1.8 ms. Thus, it is possible that the monosynaptic neural projection from the VNC to the RVLM is involved in the vestibulo-sympathetic reflex (Tables 1, 2).

**Table 1:** Baseline data of arterial pressure and heart rate in Fig. 4

	1 Hz	5 Hz	10 Hz	20 Hz	30 Hz	40 Hz	Hold
Baseline data of arterial pressure							
Control	105.7 ± 2.2	106.5 ± 1.9	105.5 ± 1.7	106.7 ± 1.3	107.3 ± 1	107.9 ± 0.7	108.3 ± 1
ChR2	105.3 ± 1.4	105.5 ± 1.6	107.3 ± 1.5	106.6 ± 1.6	105.7 ± 1.7	105.8 ± 1.4	107 ± 1.7
eArchT	105.1 ± 2.9	105.3 ± 2.2	106.2 ± 2.3	105.6 ± 2.1	105.9 ± 2.3	105.9 ± 1.3	105.8 ± 1.6
Baseline data of heart rate							
Control	347 ± 13.8	346.5 ± 12.7	344.7 ± 13.2	345.1 ± 11.4	346.3 ± 11.1	344.4 ± 11.2	345.1 ± 11.7
ChR2	345 ± 6.5	337.2 ± 6.6	350.1 ± 6.7	346.2 ± 7.2	351.6 ± 8.6	362.2 ± 10.3	361.1 ± 11
eArchT	322 ± 10.2	324.8 ± 12.6	331 ± 10.7	330.8 ± 8.9	329.8 ± 10.1	371.2 ± 10.4	369.8 ± 12.3

**Table 2:** Baseline data of arterial pressure and heart rate in Fig. 9

	Pre-HG	HG-D1	HG-D3	HG-D5
Arterial pressure	109.2 ± 1.7	111 ± 1.1	110.8 ± 3.8	100.9 ± 2.5
Heart rate	351.3 ± 12.9	328 ± 3.2	373.4 ± 7	392.9 ± 6.9

### Decrease in responsiveness of the neurons in the VNC induced by hypergravity exposure

**Fig. 9:**
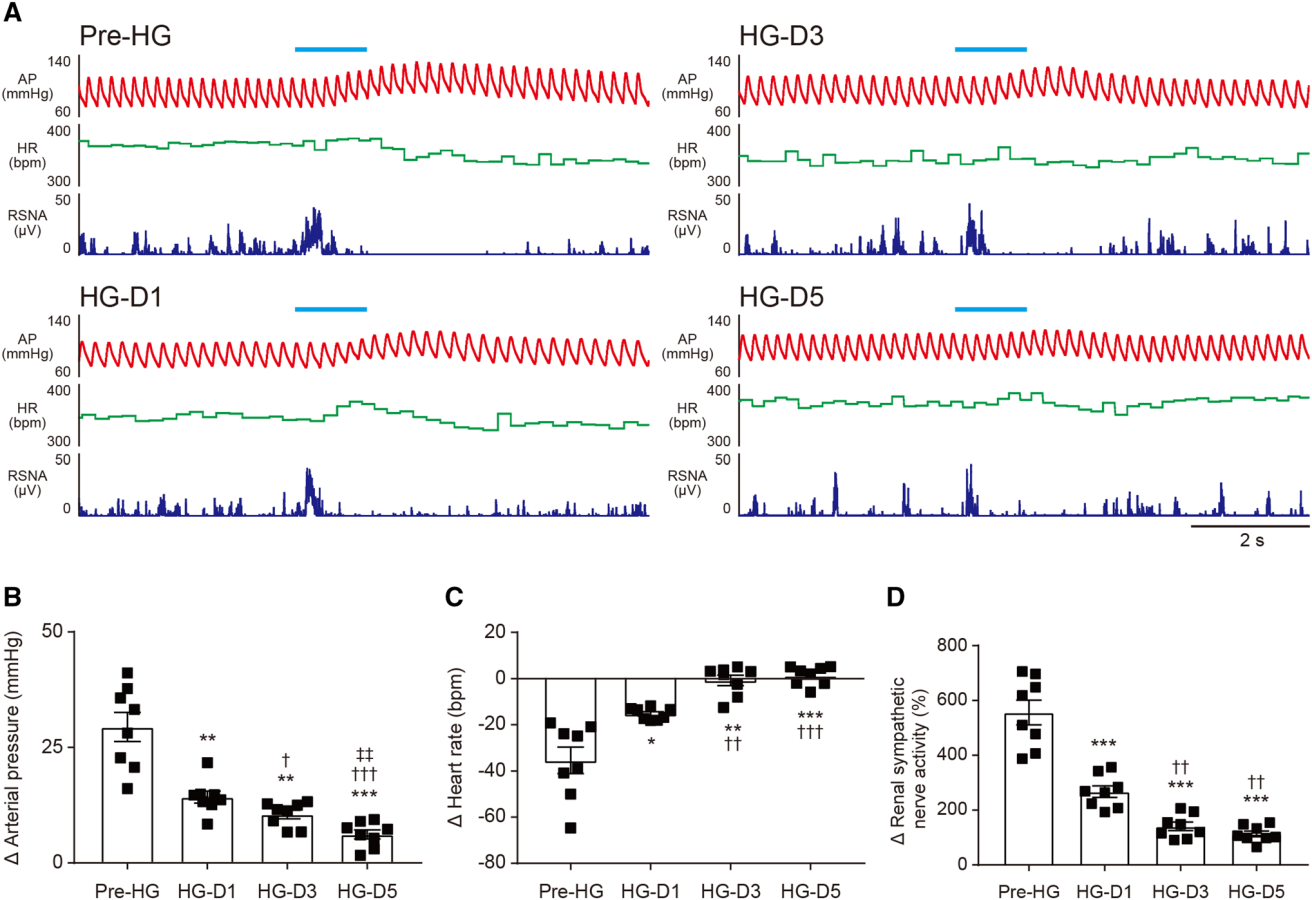
**a** Effect of excitatory photostimulation of CAMK2-expressing neurons in the vestibular nucleus complex (VNC) on the arterial pressure (AP), heart rate (HR), and renal sympathetic nerve activity (RSNA) before and during (1st, 3rd, and 5th day) exposure to a hypergravity (2 g) environment in a conscious rat. The duration of the photostimulation is 1 s. **b**–**d** Summarized data of the change in AP (**b**), HR (**c**), and RSNA (**d**) induced by excitatory (ChR2) photostimulation of the CAMK2-expressing neurons in the VNC before and during 2 g loading in conscious rats. The duration of the photostimulation is 1 s. A two-way ANOVA with Tukey’s multiple comparisons test was applied; *F*(7, 21) = 50.8, *P* < 0.0001 (**b**), *F*(7, 21) = 25.52, *P* = 0.0006 (**c**) and *F*(7, 21) = 67.45, *P* < 0.0001 (**d**). * vs. Pre-HG, ^†^ vs. HG-D1, ^‡^ vs. HG-D3. For all statistical analyses, single, double, or triple significance symbols indicate *P* < 0.05, *P* < 0.01, or *P* < 0.001, respectively

We have demonstrated that the sensitivity of the vestibulo-sympathetic reflex is attenuated by exposure to a hypergravity environment [5, 19]. We examined whether the change in responsiveness of CAMK2-expressing neurons in the VNC induced by hypergravity exposure is involved in the attenuation of the vestibulo-sympathetic reflex. The rats which were implanted with an arterial catheter and electrodes for the recording of RSNA were maintained in the hypergravity environment for 5 days. The sympathetic and cardiovascular responses induced by photostimulation were significantly suppressed even on the first day of the hypergravity loading (Fig. 9a–d). The AP response was significantly decreased dependent on the exposed day (Fig. 9b). On the other hand, the responses of the HR and RSNA were decreased by the 3rd day of the loading, and there was no difference in values between the 3rd and 5th day (Fig. 9c, d). This study demonstrated that the attenuation of the vestibulo-sympathetic reflex induced by exposure to a hypergravity environment involves at least a decrease in responsiveness of CAMK2-expressing neurons in the VNC.

## Discussion

The major findings of the present study are as follows: (1) CAMK2-expressing neurons in the VNC are involved in the balance function; (2) both excitation and inhibition of the CAMK2-expressing neurons in the VNC resulted in an increase in RSNA followed by the pressor response; (3) sympathetic and cardiovascular responses induced by the photostimulation involve the monosynaptic neural projection from the VNC to the RVLM; and (4) exposure to hypergravity environment-induced attenuation of the vestibulo-sympathetic reflex is due at least in part to a decrease in responsiveness of CAMK2-expressing neurons in the VNC.

### Vestibulo-spinal reflex through the CAMK2-expressing neurons in the VNC

Together with the cerebellum, the vestibular system contributes substantially to the stabilization of body posture during locomotion. In the present study, excitatory and inhibitory photostimulation of CAMK2-expressing neurons in the VNC resulted in body tilt, suggesting that these neurons are involved in the vestibulo-spinal reflex. The electrical stimulation of either the saccule or utricle induces the excitation of the ipsilateral vestibulo-spinal neurons [24, 25]. The vestibulo-spinal tract emerges from the VNC, especially the lateral vestibular nucleus, in the brainstem and runs unilaterally to the spinal motoneurons of the extensor muscles [26]. The present study showed that excitatory photostimulation induced body tilt to the contralateral side, suggesting that this might be a result of the contraction of the ipsilateral extensor muscles. Furthermore, the ablation of the neurons in the lateral vestibular nucleus using local injection of the diphtheria toxin resulted in both a change to the center of balance to the ipsilateral side and lower activation of the ipsilateral extensor muscle [26]. This is consistent with the data demonstrating that the inhibitory photostimulation induced body tilt to the ipsilateral side. With regard to the subsets of the neurons in the VNC, this area contains at least both glutamatergic and GABAergic/glycinergic neurons [27]. Selective expression of a reporter in excitatory neurons was observed using a 1.3 kb mouse *camkII* promoter in lentivirus pseudotyped with vesicular stomatitis virus glycoprotein [28]. However, the *camkII* promoter consistently drove gene expression in both inhibitory and excitatory neurons using adeno-associated virus (AAV) [29]. In the present study, we used AAV instead of a lentiviral vector, suggesting that opsins including ChR2 and eArchT were expressed in both the excitatory and inhibitory neurons in the VNC. It is suggested that the excitatory photostimulation of the GABAergic/glycinergic neurons in the VNC might inhibit the glutamatergic neurons in the contralateral VNC through the commissural inhibitory pathway [30], resulting in an emphasized body tilt to the ipsilateral side.

### Vestibulo-sympathetic reflex through the CAMK2-expressing neurons in the VNC

The present study showed that both excitation and inhibition of the CAMK2-expressing neurons in the VNC induced sympathoexcitation followed by the pressor response. This is consistent with our previous data demonstrating that both exposure to microgravity and hypergravity induced the pressor response through the sympathetic nervous system [5, 31]. Furthermore, these responses were not observed in anesthetized rats [32]. This is in agreement with the data showing that the photostimulation of CAMK2-expressing neurons in the VNC induced neither sympathetic/cardiovascular nor body tilt, suggesting that the vestibulo-sympathetic reflex functions only in the conscious condition. In anesthetized cats, the firing rate of the VNC neurons was changed by the stimulation using wobble rotation [33]. Since the photostimulation of RVLM neurons including Phox2b- or Dopamine beta-hydroxylase-expressing neurons induced sympathoexcitation even in the anesthetized condition [34, 35], it is possible that presynaptic inhibition can be considered in the vestibulo-sympathetic reflex under anesthesia.

CAMK2-expressing neuron-induced sympathoexcitation might occur through stimulation of the excitatory neurons as it is reported that glutamatergic, rather than GABAergic neurons, in the VNC project to the RVLM [27]. Even though the same response was observed here, the mechanism of excitation and inhibition of the CAMK2-expressing neuron-induced sympathoexcitation might be different because the form of the evoked potential in the renal sympathetic nerve was not the same (Fig. 6). In the case of excitatory photostimulation, activation of the monosynaptic neural projection from the VNC to the RVLM is involved in the sympathoexcitation because stimulation of the terminal end in the VNC neurons which project into the RVLM showed sympathoexcitation. Previous data are also supportive of this; onset latency was 40.8 ± 0.9 ms in the stimulation of RVLM neurons [35], and a longer onset latency was observed in the photostimulation of neurons in the VNC (75.1 ± 1.7 ms in cell body stimulation and 53 ± 1.8 ms in terminal end stimulation). However, the onset latency of the evoked potential in the renal sympathetic nerve was not the same in the inhibitory photostimulation of the CAMK2-expressing neurons in the VNC. The commissural inhibitory effect can be considered which increases the onset latency [30]. However, this concept is not sufficient to explain the wide skirt form of the evoked potential. The possibility of a rebound effect at the cessation of inhibitory photostimulation and/or disinhibition through the interneurons can be considered [36, 37].

### Activation of the C1 neurons in medulla through the vestibular stimulation

The present study showed that exposure to a 2 g environment significantly increased c-fos expression in C1 neurons. This was suppressed by bilateral VL, suggesting that vestibular stimulation activates C1 neurons. The C1 cells are anatomically heterogeneous, and subsets of these neurons operate as a switchboard for eliciting behaviorally appropriate patterns of autonomic responses [38]. C1 neurons are differentially activated by many types of stressors including hypoglycemia, infection or inflammation, hypoxia, nociception, and hypotension. Since the gravitational change (2 g) can be considered as one of the stressors, C1 neurons might be part of the vestibulo-cardiovascular reflex. In the RVLM, the neurons in the VNC are primarily directed toward the somata and proximal dendrites of non-catecholaminergic neurons, with minor projections to the distal dendrites of catecholaminergic cells [39]. On the other hand, some neurons in the VNC project to the nucleus of the solitary tract [40], which could activate the neurons in the RVLM [38]. Accordingly, it is possible that C1 neuron activation by vestibular stimulation is a result of an indirect pathway.

Present study showed that CAMK2-expressing neurons in the VNC induced sympathoexcitation followed by the pressor response. However, increase in HR was not observed although sympathetic nerve activity was increased. Strong excitatory photostimulation (30 Hz, 40 Hz, and hold) significantly decreased HR (Fig. 4f). This might be due to the activation of the baroreflex because of the following reasons: (1) HR response was observed after the pressor response, and (2) the inverse relationship was observed between AP and HR response. On the other hand, we have demonstrated that excitatory photostimulation of C1 neurons induces both sympathoexcitation and activation of the vagal efferent in mice [34]. Accordingly, it is possible that the photostimulation-induced no change in HR is due to the activation of both the sympathetic and the vagal efferent nerve.

### Plastic alteration of the vestibulo-sympathetic response through the CAMK2-expressing neurons in the VNC

Previously, we reported that attenuation of the vestibulo-cardiovascular reflex occurred through exposure to a hypergravity environment. Specifically, the AP response induced by linear acceleration was suppressed in rats which were maintained in a 3 g environment for 6 days [21]. This was due to the decrease in phasic inputs of the peripheral vestibular organ because the rear-up behavior and head movement was significantly suppressed in the hypergravity environment [20, 21]. The attenuation of the vestibulo-cardiovascular reflex was rescued by electrical vestibular stimulation during hypergravity loading [21]. The present study showed that the attenuation of the vestibulo-sympathetic reflex occurs after just 1 day of 2 g loading. Furthermore, this attenuation is due at least in part to a decrease in the responsiveness of CAMK2-expressing neurons in the VNC. The AP response induced by other stressors, with the exception of vestibular stimulation such as a loud noise and an air jet, was not changed by hypergravity loading [5], suggesting that the attenuation of the vestibulo-sympathetic reflex is due to the plastic alteration of the CAMK2-expressing neurons in the VNC rather than the neurons in the RVLM. Furthermore, this can explain that 2 g loading does not induce the saturation of the sympathetic nerve activity; i.e., there is a remaining capacity to induce sympathoexcitation. Since a change in gene expression occurred in the vestibular ganglion upon 2 g loading in rats [23], a decrease in responsiveness of CAMK2-expressing neurons in the VNC might be due to the influence of upstream signals. This should be examined in future studies.

## Conclusion

In conclusion, we clarified that CAMK2-expressing neurons in the VNC participate in both balance function and the sympathetic/cardiovascular response. The monosynaptic neural projection from the VNC to the RVLM is involved in the vestibulo-sympathetic pathway. Furthermore, the plasticity of the vestibulo-sympathetic response induced by gravitational change is due to a decrease in responsiveness of CAMK2-expressing neurons in the VNC. These neurons can be a target for vestibular training of elderly people and astronauts to prevent vestibular-related autonomic dysfunction.

### Electronic supplementary material

Below is the link to the electronic supplementary material.

Supplementary material 1 (MPG 2000 kb)
